# Germline Mutations in *MAP3K6* Are Associated with Familial Gastric Cancer

**DOI:** 10.1371/journal.pgen.1004669

**Published:** 2014-10-23

**Authors:** Daniel Gaston, Samantha Hansford, Carla Oliveira, Mathew Nightingale, Hugo Pinheiro, Christine Macgillivray, Pardeep Kaurah, Andrea L. Rideout, Patricia Steele, Gabriela Soares, Weei-Yuarn Huang, Scott Whitehouse, Sarah Blowers, Marissa A. LeBlanc, Haiyan Jiang, Wenda Greer, Mark E. Samuels, Andrew Orr, Conrad V. Fernandez, Jacek Majewski, Mark Ludman, Sarah Dyack, Lynette S. Penney, Christopher R. McMaster, David Huntsman, Karen Bedard

**Affiliations:** 1 Department of Pathology, Dalhousie University, Halifax, Nova Scotia, Canada; 2 Department of Pathology and Laboratory Medicine, University of British Columbia, Vancouver, British Columbia, Canada; 3 Expression Regulation in Cancer Group, IPATIMUP, Institute of Molecular Pathology and Immunology of the University of Porto & Medical Faculty of the University of Porto, Porto, Portugal; 4 Department of Ophthalmology and Visual Sciences, Dalhousie University, Halifax, Nova Scotia, Canada; 5 Medical Genetics, IWK Health Centre, Halifax, Nova Scotia, Canada; 6 Center of Medical Genetics Jacinto de Magalhães, Porto Hospital Center, Porto, Portugal; 7 Division of Anatomical Pathology, Department of Pathology, Queen Elizabeth II Health Science Center and Dalhousie University, Halifax, Nova Scotia, Canada; 8 Queen's Family Health Team, Kingston, Ontario, Canada; 9 Department of Biostatistics, Princess Margaret Cancer Centre, Toronto, Ontario, Canada; 10 Centre de Recherche du CHU Ste-Justine and Department of Medicine, University of Montreal, Montreal, Quebec, Canada; 11 Department of Pediatrics, Dalhousie University, Halifax, Nova Scotia, Canada; 12 Department of Human Genetics, McGill University, Montreal, Québec, Canada; 13 Oncogenetics Service, Institute of Medical Genetics, Meir Medical Center, Kfar Saba, Israel; 14 Department of Pharmacology, Dalhousie University, Halifax, Nova Scotia, Canada; University of Washington, United States of America

## Abstract

Gastric cancer is among the leading causes of cancer-related deaths worldwide. While heritable forms of gastric cancer are relatively rare, identifying the genes responsible for such cases can inform diagnosis and treatment for both hereditary and sporadic cases of gastric cancer. Mutations in the E-cadherin gene, *CDH1*, account for 40% of the most common form of familial gastric cancer (FGC), hereditary diffuse gastric cancer (HDGC). The genes responsible for the remaining forms of FGC are currently unknown. Here we examined a large family from Maritime Canada with FGC without *CDH1* mutations, and identified a germline coding variant (p.P946L) in mitogen-activated protein kinase kinase kinase 6 (*MAP3K6*). Based on conservation, predicted pathogenicity and a known role of the gene in cancer predisposition, *MAP3K6* was considered a strong candidate and was investigated further. Screening of an additional 115 unrelated individuals with non-*CDH1* FGC identified the p.P946L *MAP3K6* variant, as well as four additional coding variants in *MAP3K6* (p.F849Sfs*142, p.P958T, p.D200Y and p.V207G). A somatic second-hit variant (p.H506Y) was present in DNA obtained from one of the tumor specimens, and evidence of DNA hypermethylation within the *MAP3K6* gene was observed in DNA from the tumor of another affected individual. These findings, together with previous evidence from mouse models that *MAP3K6* acts as a tumor suppressor, and studies showing the presence of somatic mutations in *MAP3K6* in non-hereditary gastric cancers and gastric cancer cell lines, point towards *MAP3K6* variants as a predisposing factor for FGC.

## Author Summary

The underlying genetic mutations involved in 60% of inherited gastric cancer cases remain unknown. Here we present a large, extended pedigree with familial gastric cancer and an association in part of the family with a mutation in *MAP3K6*. The conservation, predicted pathogenicity of the variant, tissue distribution, and known function of *MAP3K6* made this a strong candidate that warranted further investigation. Examination of an additional 115 unrelated probands identified additional mutations in *MAP3K6*, including a truncating mutation.

## Introduction

Gastric cancer is the second leading cause of cancer-related death worldwide with 738,000 deaths per year [1]. Primary treatment consists of surgical resection of the tumor and may be followed by chemotherapy and/or radiotherapy. The 5-year survival rates after surgical resection are high if the disease is detected early (71% for stage 1A), however, they drop off quickly when the diagnosis is made at later stages (46% stage IIA, 20% stage IIIA, 4% stage IV) (National Cancer Institute's SEER database, October 2013). Unfortunately, because early symptoms of gastric cancer closely resemble other diseases, detection often does not occur until advanced stages have already been reached [2].

Classically, gastric cancer has been divided into two types: intestinal and diffuse [3]. The intestinal form occurs spontaneously and is most often found in elderly individuals, while the diffuse form often occurs in younger individuals and can be associated with a family history of gastric cancer. Populations with higher prevalence of chronic *Helicobacter pylori* infection tend to have higher gastric cancer burdens [4]. The majority of gastric cancers (90%) are sporadic, but approximately 10% show familial clustering [5]. Only 1% to 3% are caused by a hereditary syndrome, as opposed to environmental factors such as shared dietary practices [5]. The most well established familial form of gastric cancer is hereditary diffuse gastric cancer (HDGC [MIM 137215]), where approximately 40% of cases are attributed to germline mutations in the E-cadherin encoding gene, *CDH1* [6]–[9].

We ascertained a large family from Maritime Canada with a history of Familial Gastric Cancer (FGC) displaying an apparent autosomal dominant pattern of inheritance, but bearing no variants in the coding region of the *CDH1* gene. While the family displays many features typical of HDGC, there was diversity in the clinical presentation within the family as well as an advanced age of onset, therefore we have opted to simply refer to the condition as FGC over the more stringently defined HDGC. Genomic mapping of shared inherited regions among affected family members, followed by whole-exome sequencing, led to the identification of a germline single nucleotide variant (SNV) in mitogen-activated protein kinase kinase kinase 6 (*MAP3K6*, *ASK2*, *MAPKKK6*, *MEKK6*, ENSG00000142733), a gene encoding a member of the serine/threonine protein kinase family. Several *in silico* methods predicted the SNV in MAP3K6 to be damaging to the protein, and previous studies with *MAP3K6* deficient mice [10], as well as the occurrence of mutations in this gene in both primary gastric cancer tumors and gastric cancer cell lines [11], were consistent with mutations in the *MAP3K6* gene being the causative mutation. Sequencing of DNA isolated directly from a fixed tumor specimen of one individual demonstrated the presence of a *de novo* second-hit variant in *MAP3K6*. Screening of an additional 115 unrelated FGC samples, also negative for *CDH1* mutations, revealed five individuals with four additional SNVs in *MAP3K6* that were also predicted to be pathogenic, as well as an unrelated individual with the SNV identified in the family from Maritime Canada. The age of onset varied among *MAP3K6* SNV carriers in the five families, and one had not developed cancer even at late stage of life, suggesting incomplete penetrance. This is the first report of a heritable cancer resulting from SNVs in *MAP3K6*.

## Results

### Clinical and Pathological Assessment

**Figure 1:**
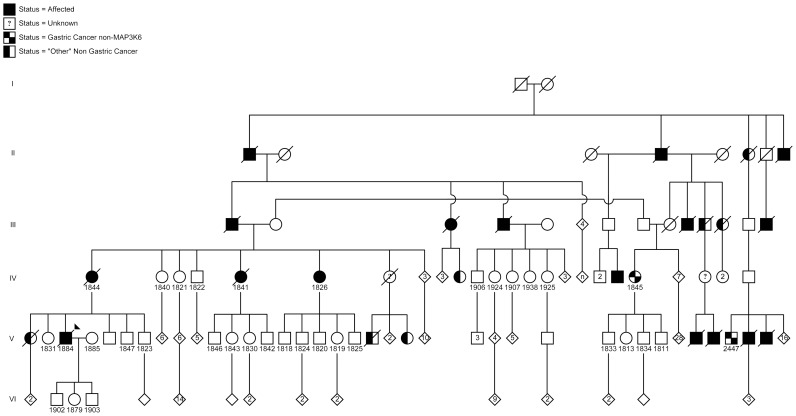
Pedigree of the Maritime Canadian family. Clinically affected individuals are indicated with shaded symbols. Individuals for whom DNA samples were collected are indicated by a number. Individuals shaded within 2 quarter sections were affected but were negative for the MAP3K6 mutation. Individuals shaded on one half had another, non-gastric, cancer. Generations I–VI are indicated.

We ascertained a large Maritime Canadian family of European descent in the course of routine clinical assessment in the Hereditary Cancer Clinic as part of Maritime Medical Genetics Service at the IWK Health Centre in Halifax, Nova Scotia, Canada (Figure 1). Saliva, blood, or formalin-fixed paraffin-embedded (FFPE) samples were obtained from 6 family members with gastric cancer, as well as 27 unaffected relatives, and one married-in individual. No consanguinity was suspected in this pedigree.

**Figure 2:**
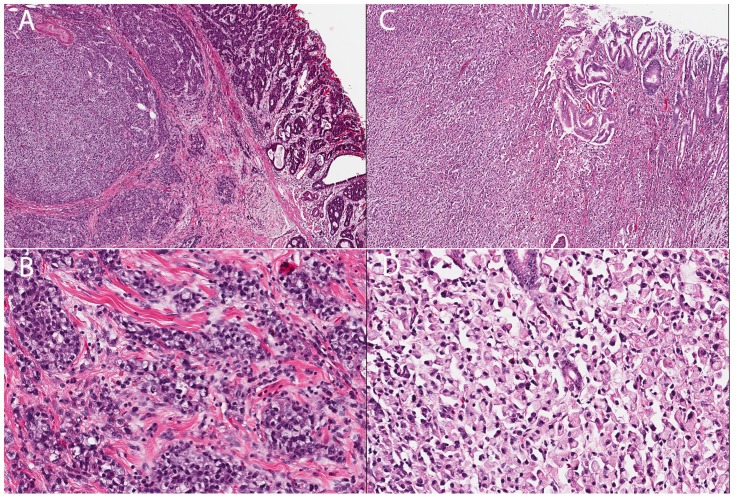
Histology of two gastric cancer patients from the Maritime Canadian family. **A**) Patient 1826. Section shows a tumor composed of solid and glandular components (H&E, 40×). **B**) The tumor cells show signet ring cell morphology in solid areas (H&E, 200×). **C**) Patient 1884. Section shows sheet of tumor cells infiltrated lamina propria and submucosa (H&E, 40×). **D**) The tumor entirely consists of signet ring cells (H&E 200×).

The proband, affected individual 1884, was diagnosed with metastatic gastric carcinoma and underwent a total gastrectomy at age 51. Pathological examination revealed a poorly differentiated adenocarcinoma arising in the antrum of the stomach in a background of intestinal metaplasia and chronic gastritis. The tumor was composed of a sheet of signet ring cells (Figure 2C and D). The carcinoma penetrated through the entire thickness of the muscularis propria involving the serosal layer. No evidence of *H. pylori* was seen.

The proband's maternal aunt, individual 1826, was diagnosed with gastric carcinoma at age 80 and underwent a partial gastrectomy. Pathological examination revealed a moderately to poorly differentiated adenocarcinoma invading into muscularis propria (Figure 2A). The tumor was predominantly composed of cohesive nests of neoplastic cells with occasional glandular formation. Tumor cells with signet ring cell forms were seen in solid areas (Figure 2B). The gastric mucosa adjacent to the tumor showed focal intestinal metaplasia without evidence of *H. pylori*.

At age 76 a stomach biopsy of another maternal aunt to the proband, individual 1841, was reported to have a moderately differentiated adenocarcinoma with glandular formation. *H. pylori* was identified in the background gastric mucosa. A small biopsy section was available for re-examination. Although this sample was too small for a complete classification, it showed cohesive nests of neoplastic cells with small foci of glandular formation in keeping with a poorly differentiated adenocarcinoma. There were some tumor cells in the sample showing clear cytoplasm, but these could not be definitively classified as signet ring cells.

A stomach biopsy at age 82 of patient 1844, the proband's mother, showed a poorly differentiated adenocarcinoma with signet ring features. The background mucosa showed evidence of *H. pylori* and patchy intestinal metaplasia.

Patient 1845, a first-cousin-once-removed to the proband, was diagnosed at age 59 with an undifferentiated carcinoma without signet ring features. The tumor was associated with dense lymphoid infiltrate and was best classified as lymphoepithelial carcinoma. *H. pylori* was not seen in the adjacent normal mucosa. The tumor was positive for two intronic variants in *CDH1* both of which are expected to be benign (NM_004360.3:c.688−83G>A and c.2439+52G>A).

Patient 2447, a third cousin, was diagnosed at age 44 with a poorly differentiated adenocarcinoma without signet ring cell features. The adjacent gastric mucosa showed extensive intestinal metaplasia. There was no evidence of *H. pylori*.

**Figure 3:**
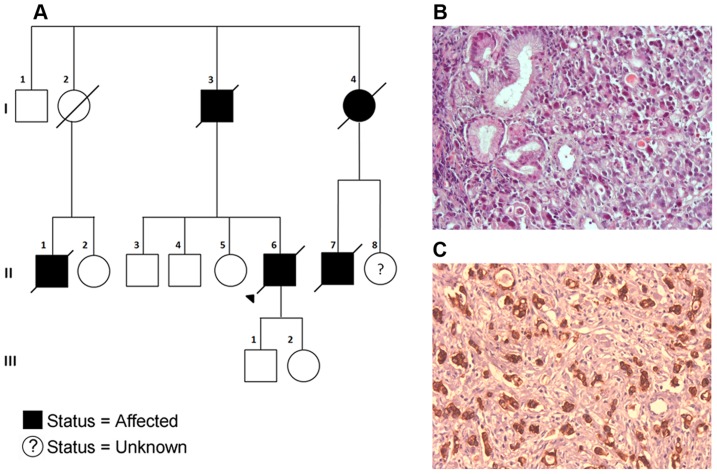
A) Pedigree of the Portuguese Familial Gastric Cancer family. Affected individuals are shaded in black with the sequenced proband indicated with a triangle. Deceased individuals are marked with a strike-through. Generations I–III are indicated. **B**) Tumor cells showing signet ring cell morphology (H&E, 200×). **C**) Tumor cells retaining E-cadherin protein expression (IHC analysis performed with the rabbit anti-E-cadherin Antibody (24E10 Cell Signaling, MA, USA), according to manufacturer's instructions, 200×).

Following screening of a panel of 115 probands with non-*CDH1* familial gastric cancer, an unrelated family from Portugal was added to our study (Figure 3A). Individual II-6 was diagnosed with gastric cancer at age 62, having poorly differentiated adenocarcinoma of the stomach and the presence of signet ring cells. Immunohistochemistry analysis showed positive membranous staining of E-cadherin in neoplastic cells (Figure 3C), including signet ring cells (Figure 3B). The related individuals I-4, II-1, and II-7 were diagnosed with gastric cancer (histology details unknown) at ages 53, 62 and 52 respectively. All four individuals in the Portuguese pedigree died from the disease within 5 years of diagnosis in this family. In the Maritime Canadian family, 1884, 1844 and 1841 died from the disease within one year of diagnosis.

The gastric cancer described for patients 1845 and 2447 had no signet rings observed and was diagnosed at an earlier average age (52 versus 72, although the proband was diagnosed at age 51). Based on differences in histology, particularly the lack of signet ring cells in 1845 and 2447 compared to the other affected individuals, it is possible that the disease in these two individuals represents a distinct condition. Alternatively it is possible that the family is displaying a more complex phenotypic pattern being driven by two (or more) genes.

### Molecular Mapping and Exclusion of Known and Candidate Genes

Although 30–40% of HDGC cases are attributable to mutations in *CDH1*, no mutations in protein-coding exons of *CDH1* were found in affected individuals from the Maritime Canadian family. To identify the pathogenic loci in this family, high density SNP-genotyping using Illumina arrays was performed on five affected individuals: the proband's mother (1844), two affected maternal aunts (1826 and 1841), and two distant cousins (1845 and 2447) as well as several related individuals with no reported incidence of cancer whose affection status was treated as unknown (1907, 1924, 1821, and 1822). For all individuals except 1845 genotyping data was available at 2.5 million markers. Individual 1845 had previously been genotyped at a density of 660K, and was not able to be re-genotyped at the higher density. No DNA suitable for SNP genotyping was obtained from the FFPE sample of the proband (1884). Using these data, we performed both non-parametric and parametric linkage analysis using Merlin [12]. Given the late age of onset in many affected family members, the penetrance in the Maritime pedigree is unknown. In order to be conservative in identifying genomic regions of interest, two dominant penetrance models (50% and 99% penetrance) using affected individuals 1826, 1841, 1844, and 2447 (and individuals 1907, 1924, 1821, and 1822 with unknown affection status) were used. Genomic regions identified under parametric linkage analysis were generally consistent with one another regardless of the penetrance parameter chosen (Table 1). The analysis was repeated with individual 2447 treated as unknown to analyze just the reduced pedigree where 2447 and 1845 were treated as potential phenocopies (Table 2). This resulted in lower overall LOD scores for all regions identified, as well as more and larger regions on average, encompassing a larger portion of the genome.

**Table 1:** Regions with LOD>1 from pedigree-wide parametric linkage analyses using Merlin.

Penetrance: 99%					
Chr	Max LOD	Start SNP	Start bp	End SNP	End bp
1	1.0678	rs1409162	71,368,758	rs871664	94,609,478
3	1.7039	rs938087	194,291,805	rs2686097*	197,304,279
7	1.6951	rs4722077	22,011,292	rs10281171	36,807,381
10	1.5943	rs7923552	21,687,123	rs10508881	44,541,565
15	1.5321	rs17626899	94,215,925	rs11073574	98,306,216
16	1.1006	rs9302859	7,989,791	rs4785367	49,956,194
17	1.6395	rs1269480	49,815,361	rs9897212	60,190,630
20	1.6937	rs4501008	51,433,681	rs6027887	59,449,041

Penetrance: 50%					
Chr	Max LOD	Start SNP	Start bp	End SNP	End bp
3	1.4762	rs938087	194,291,805	rs2686097*	197,304,279
7	1.4597	rs4722077	22,011,292	rs10281171	36,807,381
10	1.3718	rs9645525	18,620,950	rs2153332	45,731,944
15	1.3014	rs17626899	94,215,925	rs11073574	98,306,216
16	1.471	rs8055473	9,798,539	rs4785367	49,956,194
17	1.4055	rs1269480	49,815,361	rs9897212	60,190,630
20	1.4584	rs4501008	51,433,681	rs6027887	59,449,041

Genomic intervals and associated LOD scores are shown under dominant models with 50% or 99% penetrance. Regions are defined by their 1-LOD support interval. Base pair positions are from hg19. An asterisk indicates the SNP was the first or last analyzed marker on the chromosome.

**Table 2:** Regions with LOD>0.5 from parametric linkage analyses using Merlin when individual 2447 is treated as unknown (sub-pedigree).

Penetrance: 99%					
Chr	Max LOD	Start SNP	Start bp	End SNP	End bp
1	0.5692	rs1702312	25,364,652	rs2039943	102,297,845
2	0.5692	rs6718709	5,340,502	rs7562738	104,060,568
3	0.5692	rs2363970	190,873,984	rs2686097*	197,304,279
5	0.5692	rs4957023*	336,952	rs1604563	3,718,709
5	0.5692	rs1437118	94,301,042	rs889056	157,039,737
6	0.5692	rs6454657	88,571,309	rs3736746	144,259,554
7	0.569	rs12530679	106,632,113	rs850398	145,130,880
8	0.5692	rs12156116	138,551,517	rs750472*	145,701,453
9	0.5692	rs1454637	2,596,569	rs912670	115,673,030
10	0.5692	rs7906313*	766,105	rs10762712	54,112,284
12	0.5692	rs10846635	124,728,014	rs7296889*	133,054,656
13	0.5692	rs7992848	32,800,816	rs9571607	67,204,131
14	0.5692	rs4497606*	20,669,142	rs225904	30,454,892
16	0.5692	rs9302859	7,989,791	rs8046479	83,227,014
17	0.5692	rs7215862	14,282,778	rs9897212	60,190,630
20	0.5691	rs4813237	16,635,038	rs6027887	59,449,041
22	0.5586	rs2337542	47,758,290	rs2688098	49,682,956
23	0.5692	rs6567564*	3,510,277	rs5945157*	152,901,900

Penetrance: 50%					
Chr	Max LOD	Start SNP	Start bp	End SNP	End bp
1	0.5163	rs11249206	25,277,982	rs2208963	104,529,736
2	0.5163	rs6718709	5,340,502	rs7562738	104,060,568
3	0.5163	rs2363970	190,873,984	rs2686097*	197,304,279
5	0.5163	rs4957023*	336,952	rs580641	4,041,154
5	0.5163	rs1437118	94,301,042	rs17608675	157,340,772
6	0.512	rs6454657	88,571,309	rs3736746	144,259,554
7	0.5162	rs12530679	106,632,113	rs850398	145,130,880
8	0.5163	rs12156116	138,551,517	rs750472*	145,701,453
9	0.5163	rs1454637	2,596,569	rs1570756	115,775,168
10	0.5163	rs7906313*	766,105	rs10762712	54,112,284
12	0.5163	rs4765078	124,646,827	rs7296889*	133,054,656
13	0.5163	rs9600625	31,470,643	rs9571607	67,204,131
14	0.5163	rs4497606*	20,669,142	rs225904	30,454,892
16	0.5163	rs9302859	7,989,791	rs8046479	83,227,014
17	0.5163	rs7215862	14,282,778	rs9897212	60,190,630
20	0.5162	rs4813237	16,635,038	rs6027887	59,449,041
22	0.5063	rs2337542	47,758,290	rs2688098	49,682,956
23	0.5163	rs6567564*	3,510,277	rs5945157*	152,901,900

Genomic intervals and associated LOD scores are shown under dominant models with 50% or 99% penetrance. Regions are defined by their 1-LOD support interval. Base pair positions are from hg19. An asterisk indicates the SNP was the first or last analyzed marker on the chromosome.

We also performed non-parametric linkage (NPL), a method with fewer underlying assumptions about the underlying inheritance model, using affected individuals 1826, 1841, 1844, and 2447 (pedigree-wide) or with the removal of 2447 as a potential phenocopy by specifying them to be of unknown status (sub-pedigree). Pedigree-wide genomic intervals were mostly consistent with those identified using the two parametric models (Table 3). Exclusion of 2447 resulted in a lower overall maximum score (1.204), which was found on several intervals throughout the genome (Table 3).

**Table 3:** Genomic intervals with NPL>2 or >1 by non-parametric linkage analysis using the S_all_ scoring function, under the exponential model of Kong and Cox. Results including 2447 (NPL>2) or excluding 2447 (NPL>1) are shown.

Including 2447						
Chr	MaxNPL	p-value	start SNP	start bp	end SNP	end bp
1	2.001	0.0012	rs4607942	76,750,428	rs472908	94,487,354
3	2.709	0.0002064	rs938087	194,291,805	rs2686097*	197,304,279
10	2.593	0.0002743	rs7923552	21,687,123	rs12781751	31,872,826
10	2.482	0.0003609	rs7911097	36,651,214	rs10508881	44,541,565
16	2.703	0.0002094	rs8055473	9,798,539	rs4785367	49,956,194

Excluding 2447						
Chr	MaxNPL	p-value	start SNP	start bp	end SNP	end bp
1	1.204	0.009266	rs3767879	26,070,909	rs545247	29,174,044
1	1.204	0.009266	rs545247	29,174,044	rs9326132	43,471,182
1	1.204	0.009266	rs11206577	55,860,561	rs11578884	70,282,875
1	1.204	0.009266	rs11578884	70,282,875	rs472908	94,487,354
2	1.204	0.009266	rs11675383	20,882,949	rs2374292	41,849,795
3	1.204	0.009266	rs938087	194,291,805	rs2686097*	197,304,279
5	1.204	0.009266	rs4957023*	336,952	rs2922062	2,618,844
5	1.204	0.009266	rs10065787	131,436,486	rs853160	142,559,092
6	1.204	0.009266	rs6901395	89,432,362	rs11153525	115,231,965
6	1.195	0.009491	rs11153525	115,231,965	rs4382328	123,022,496
7	1.204	0.009266	rs7777931	135,016,454	rs12703526	143,107,588
8	1.204	0.009266	rs7007907	139,914,637	rs2233230	140,943,503
9	1.204	0.009266	rs7025491	37,387,177	rs11138454	82,623,065
9	1.155	0.01055	rs11138454	82,623,065	rs7849075	86,149,610
10	1.204	0.009266	rs7906313*	766,105	rs10905707	10,321,874
10	1.204	0.009266	rs2446580	14,005,536	rs1774221	30,254,563
12	1.204	0.009266	rs7135277	128,737,601	rs7296889*	133,054,656
13	1.204	0.009266	rs2281810	43,461,643	rs8002829	49,951,142
14	1.204	0.009266	rs4497606*	20,669,142	rs761874	23,596,320
16	1.204	0.009266	rs150583	21,712,743	rs4620978	62,192,704
17	1.204	0.009266	rs3764866	26,800,204	rs3916033	44,889,703
20	1.204	0.009266	rs6028809	38,663,214	rs4142387	42,017,672
22	1.182	0.009834	rs133651	48,717,026	rs9617453	49,034,053
23	1.204	0.009266	rs6567564*	3,510,277	rs2188385	9,923,646

An asterisk indicates the SNP was either the first or last marker on the chromosome. Base pair positions are from hg19.

Genomic intervals identified in this manner were used for filtering the exome sequencing data to identify potential causative mutations. To be broad in identifying possible causative mutations, in both the pedigree-wide and sub-pedigree case the intervals from the respective parametric and non-parametric analyses were combined. For the pedigree-wide analyses this was the union of intervals described in Table 1 along with the appropriate intervals in Table 3 (including 2447) and for the sub-pedigree analysis the union of intervals found in Table 2 with appropriate intervals in Table 3 (excluding 2447).

### Whole Exome Sequencing

We next performed whole-exome sequencing on two of the affected maternal aunts to the proband (1826 and 1841) and the affected third cousin (2447). We prioritized and filtered variants based on their frequency among European-descent populations (<2% and a stricter filter at <1%) from the 1000 Genomes and Exome Sequencing Project datasets as well as other exomes sequenced at the same sequencing provider, location within a genomic region of interest, and the functional consequence of the mutation (altering the protein coding sequence or splice site of at least one protein coding transcript). Variants of interest were then sequenced by Sanger sequencing in other affected individuals. Variant filtration based on genomic intervals was performed separately for each hypothesis (whole-pedigree and reduced-pedigree) (Tables S1 and S2). In addition to the identification and filtration of genetic variants, we assessed the sequencing depth of coverage of exons (defined by the Consensus CDS set) within genomic regions of interest and across individual exome sequencing results. Further, we searched for potentially shared variants that were “masked” by coverage issues. For all variants observed in one or more exomes, if no variant was observed in the remaining exome(s), we evaluated whether that was due to low coverage or coverage gaps within the exon. For variants where this was true we filtered using standard criteria (as above). Using these filtering criteria, several variants with low MAF and potentially having an effect at the protein-coding level were observed in the pedigree-wide genomic regions of interest that had been identified by parametric linkage analysis; however, none were present in all affected individuals. Further no “masked” candidate variants were identified by the same criteria.

We considered the possibility that individuals 1845 and 2447 have a distinct clinical condition, and examined the variants shared among the proband and immediate family. Using the same filtering criteria as above, but using only the exomes from individuals 1826 and 1841, a total of 127 variants were identified. Stricter filtering for rare variants (MAF <1%) reduced this to number to 85 (Table S2). A subset of these variants, based on a combination of factors (mutations in COSMIC [13], predicted effect of the mutation, conservation of the encoded amino acid, literature review, known expression patterns in normal tissues and tumors, disease phenotypes associated with the gene) were sequenced for follow-up in the proband and their mother. A variant in *MAP3K6* (Chr1, NM_004672) was of particular interest. A mutation identified in *MAP3K6* (c.[2837C>T];[ = ], p.P946L) was considered a strong candidate based on the known associations of other MAP kinases with cancer, and several publications elucidating a role for MAP3K6 in tumorigenesis [10], [11], [14], [15]. This variant has been reported previously (rs141787524) with a minor allele frequency of 0.7% in the 1000 Genomes Project (European descent group) and a frequency of 0.4% in the European-American population (Exome Variant Server (NHLBI GO Exome Sequencing Project (ESP): http://evs.gs.washington.edu/EVS [Accessed October, 2013]). It was seen as a heterozygous variant in 11 (of 1532) other exomes sequenced at the Genome Quebec Innovation Centre, corresponding to a MAF of 0.36%.

This SNV was present in four affected individuals in the Maritime family (1884, 1826, 1844, 1841), of which three clearly showed the presence of signet ring cells. Only a small punch biopsy was available for the maternal aunt, 1841, therefore we were not able to definitively confirm the presence or absence of signet ring cells. The *MAP3K6* SNV was also present in five of the 27 currently unaffected relatives sampled, and it was not present in the married-in relative. One of the carriers was homozygous for the SNV and was over 80 years old with no reported cancer. Although no consanguinity was reported in the family, and no evidence of copy number variation was observed in the SNP genotype data, this individual was also homozygous for a 10 Mb region encompassing the locus. The remaining carriers ranged in age from 33 to 51, and as the age of onset of the cancer was generally later, their status was considered “unknown”. Both individuals, 1845 and 2447, with the phenotypically distinct gastric cancer were negative for the *MAP3K6* SNV.

### Somatic Variants within the Tumor

We next used the DNA isolated from a tumorous section of the FFPE sample of the affected *MAP3K6* SNV carrier 1884 (the proband) to screen for additional somatic SNVs or loss of heterozygosity (LOH) within the tumor itself. In addition to the p.P946L variant, we identified a novel SNV in the *MAP3K6* gene at position c.[1516C>T] leading to an amino acid change p.H506Y (Table 4), and were able to infer that the SNV was somatically acquired based on sequence data from the spouse and children.

**Table 4:** Summary information for each of the germline and somatic mutations found in *MAP3K6*.

Position (hg19)	Amino Acid Change	HGVS (NM_004672)	dbSNP ID	MAF (1000G/EVS) (%)
Chr1: ;;;27690792	D200Y	c.598G>T	rs41291098	0.4/0.4
Chr1: ;;;27690770	V207G	c.620T>G	rs182712391	-/0.01
Chr1: ;;;27688258	H506Y^a^	c.1516C>T	-	-/-
Chr1: ;;;27684750	P946L	c.2837C>T	rs141787524	0.7/0.4
Chr1: ;;;27684715	P958T	c.2872C>A	rs75893867	-/-
Chr1: ;;;27685238-27685239	p.F849Sfs*142	c.2544delC	rs34008139	-/-

a The H506Y mutation is a somatic second-hit observed in FFPE tumor tissue from patient 1884.

### Verification in Unrelated FGC Cases

**Figure 4:**
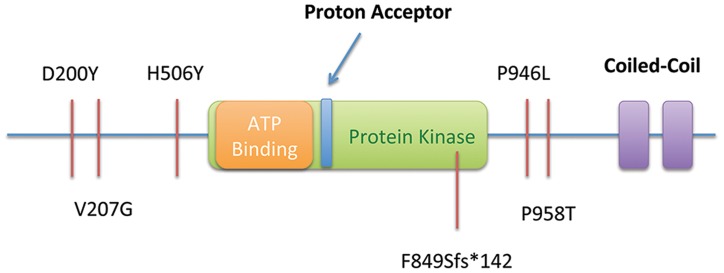
Summary of SNVs observed in the Maritime sub-pedigree (Proband, Mother, Maternal Aunts) and in 115 probands from an additional screened cohort, including a frameshift-truncating mutation Portuguese pedigree (F849Sfs*142). All SNVs are shown in relation to the predicted functional domains of *MAP3K6*.

We screened DNA samples from an additional 115 unrelated FGC individuals using a multiplexed targeted next generation sequencing assay. Samples were from unrelated families that met international gastric cancer linkage consortium (IGCLC) criteria for hereditary diffuse gastric cancer (106), but had previously tested negative for mutation of the *CDH1* locus, or familial intestinal gastric cancer. Within this cohort, we identified five additional heterozygous SNVs in the *MAP3K6* gene (Table 4): a truncating SNV (c.[2544delC], p.F849Sfs*142), three missense SNVs (c.[2872C>A], p.P958T; c.[598G>T], p.D200Y; and c.[620T>G], p.V207G), and another individual with the p.P946L (c.[2837C>T]) variant, previously found in the Maritime family (Figure 4). Mutations in *MAP3K6* were only discovered in the individuals meeting the criteria for diffuse gastric cancer (106). The individual in this novel patient cohort carrying the p.P946L variant is thought to be unrelated to the Maritime family.

The truncating SNV was observed in a Portuguese individual with a family history of gastric cancer (Figure 3A). While this SNV has a dbSNP identifier (rs34008139), no population frequency has been associated with it from either the 1000 Genomes or Exome Variant Server projects. This SNV has also been reported in the COSMIC database [13] (somatic/germline status not specified) in a carcinoma sample of the large intestine. Histological examination of the proband's tumor showed a poorly differentiated gastric cancer with signet ring cells, retaining E-cadherin protein expression at the cell membrane (Figure 3B and 3C).

The p.D200Y variant was found in probands from two unrelated families, and has been observed within the 1000 genomes cohort (rs41291098), but is also rare with a minor allele frequency (MAF) of 0.4% in both the 1000 Genomes and Exome Sequencing Project European descent groups. The p.P958T variant (rs75893867) has no MAF reported in either the 1000 Genomes or Exome Sequencing Project European descent datasets, and was only identified in the 1000 Genomes among the Japanese cohort at a frequency of 2.2%. However, it has been identified in COSMIC (COSM99077) as a somatic mutation from a gastric carcinoma patient. The p.V207G variant has been identified in the Exome Sequencing Project European-American dataset with a MAF of 0.01%. It was not identified among European or European descent groups within the 1000 Genomes, but was observed within other population groups at a range of minor allele frequencies.

Along with *MAP3K6*, 50 additional genes previously suggested to be involved in risk for disease of the upper gastrointestinal tract were sequenced for this cohort using a custom panel-based assay (manuscript submitted). Genes for the custom MiSeq-based screen were selected based on literature review, as well as genes of interest in collaborative projects. In the cases where *MAP3K6* missense variants were identified, no other candidate variants were found.

### Second-Hit Analysis in the Tumor from the Portuguese Family

**Figure 5:**
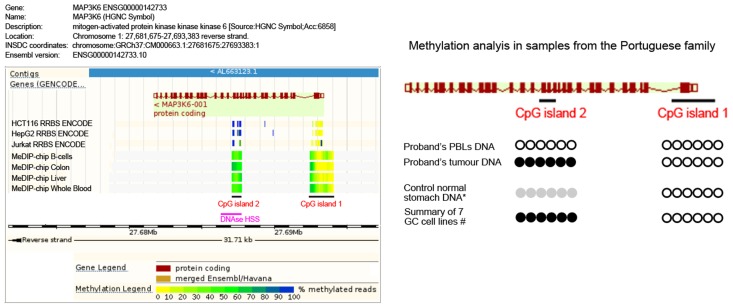
Methylation analysis of the Portuguese family. Left panel: Schematic representation of the MAP3K6 gene adapted from Ensembl genome browser (release 75). The two CpG islands analyzed are represented. CpG island 1 is mainly non-methylated for several normal tissues and cells lines represented in the scheme, while CpG island 2 displays low methylation frequency (light green) in normal tissues such as B-cells, Colon, Liver and Whole Blood, and high methylation (blue) in colon (HCT116), liver (HepG2) and blood (Jurkat) cancer cell lines. A DNase HSS predicted to harbor a promoter-associated regulatory element overlapping with CpG island 2. Right panel: For the CpG island 1, no hypermethylation was detected (white circles). For the CpG island 2, we observed complete methylation in the proband's tumor DNA (black circles) and no methylation in the PBLs' DNA. The DNA of normal gastric mucosa from controls displayed partial methylation (grey circles). All gastric cancer cell lines mimicked the full methylation observed for the tumor DNA (black circles).

FFPE tumor tissue was available for the proband from the Portuguese family carrying the p.F849Sfs*142 germline truncating mutation. Somatic mutations were excluded in the complete coding sequence and intron-exon boundaries of *MAP3K6* in the proband's tumor. Lack of LOH at the *MAP3K6* gene could also be inferred in this tumor, as both wild-type and mutant alleles could be identified at the germline mutation site (Supplementary Figure S2). We therefore searched for putative alternative inactivating mechanisms. Hypermethylation of CpG islands within gene promoters and regulatory regions is a common phenomenon leading to decreased gene expression in cancer [16]. *MAP3K6* regulation by promoter hypermethylation has been described for human bone marrow mesenchymal stem cell [17], although a correlation of hypermethylation and gene expression has not been established. We searched for *MAP3K6* CpG islands [18] and found two CpG islands, one at the promoter region and another encompassing exon 10 and part of the downstream intron (Figure 5 and Supplementary Figure S1). The downstream CpG island (CpG island 2) is near a DNase hypersensitive site predicted to harbor promoter associated features (Ensembl regulatory feature ID ENSR00000533270, Figure 5). We bisulfite-treated DNA from: the proband's peripheral blood lymphocytes (PBLs); the proband's tumor; four different normal stomach control samples, and; seven gastric cancer cell lines. For the promoter CpG island (CpG island 1), no hypermethylation was detected using two different primer sets (Figure 5 and Suppl. Figure S1). Regarding CpG island 2, we observed complete methylation for the tumor DNA and no methylation for the PBLs DNA. Interestingly, the methylation analysis at CpG island 2 in normal stomach mucosa from controls displayed a partial methylation pattern. In line with the result obtained for tumor DNA, all seven gastric cancer cell lines displayed full methylation (Figure 5 and Suppl. Figure S1).

### Pathogenicity of SNVs in *MAP3K6*

We applied a variety of *in silico* methods to predict the pathogenicity of the observed missense SNVs. Although there was no full consensus across programs (Table 5), all of the SNVs were considered deleterious by at least one program, and except p.V207G and p.P958T, the other four variants described in this report were predicted to be deleterious by at least 3 of the 7 methods. In addition, the EvoD [19] consensus prediction (based on a balanced combination of the EvoD, PolyPhen2 [20], and SIFT [21] scores) reported that three of the variants (p.D200Y, p.V207G, p.H506Y) were deleterious or likely to be deleterious. The two remaining variants, p.P946L and p.P958T, were predicted to likely be neutral changes although they were evolutionarily ultra-conserved and well-conserved (according to EvoD evolutionary rate classification) respectively; while only one of the other three variants (p.D200Y) was considered well-conserved and the other two were less-conserved. As (i) a *MAP3K6* truncating variant was found, (ii) there was a second-hit variant identified in the individual for whom FFPE tissue was examined, and (iii) all of the programs tested are designed to predict pathogenicity based on loss of function, it is likely that the variants described lead to either a decrease in function or dominant negative phenotype.

**Table 5:** Predicted pathogenicity of germline and somatic variants in *MAP3K6* observed in a Maritime Canadian family and probands from a screen of 115 FGC cases negative for *CDH1* mutations.

Amino Acid Change	PolyPhen2	PMut	PROVEAN	SIFT	EvoD	FATHMM*	MutationTaster
D200Y	Probably Damaging	Neutral	Deleterious	Damaging	Neutral	Tolerated/Passenger	Disease Causing
V207G	Benign	Neutral	Neutral	Tolerated	Deleterious	Tolerated/Passenger	Polymorphism
H506Y^a^	Possibly Damaging	Neutral	Neutral	Damaging	Deleterious	Tolerated/Passenger	Polymorphism
P946L	Probably Damaging	Pathological	Neutral	Tolerated	Neutral	Tolerated/Passenger	Disease Causing
P958T	Possibly Damaging	Neutral	Neutral	Neutral	Neutral	Tolerated/Passenger	N/A
p.F849Sfs*142	-	-	-	-	-	-	-

* FATHMM predictions for Missense Variants and Cancer-Associated Variants tools are both given.

a The H506Y mutation is a somatic second-hit observed in FFPE tumor tissue from patient 1884.

## Discussion

Here we present the first evidence that germline mutations in *MAP3K6* are linked to inherited cancer. Four individuals with gastric cancer from a Maritime Canadian family were found to carry a heterozygous variant in the *MAP3K6* gene, leading to a p.P946L amino acid change. This germline variant, located on chromosome 1, was identified in two of the three exome samples and was located within a region identified by parametric and non-parametric linkage analysis within the sub-pedigree (1845 and 2447 treated as “unknown” disease status). The significance of the *MAP3K6* variant was supported by the identification of a somatic second-hit mutation in the *MAP3K6* gene at p.H506Y present in DNA isolated directly from a tumorous section of a patient FFPE sample. Two individuals from the pedigree with gastric cancer, but with some phenotypic differences did not carry the mutation; however, no candidate variants were identified shared among all affected individuals within any region identified, by parametric linkage conducted pedigree-wide.

Screening of additional FGC families revealed five other *MAP3K6* mutations, including a p.F849Sfs*142 germline mutation observed in the Portuguese proband, which is expected to lead to protein truncation. After excluding somatic mutations and LOH, a potential second-hit mechanism was found *via* hypermethylation at an intragenic CpG island near a predicted promoter-associated regulatory element (DNAse I hypersensitive site). The relevance of methylation at this *MAP3K6* gene region could not be ascertained in terms of impact in gene expression, nevertheless the possibility of acting as a possible second-hit inactivation (partial or complete) mechanism is raised, due to the results obtained in normal stomach and cancer cell lines. If so, this may well represent another example of the increasingly recognized concept that DNA methylation in the gene body is not just a passive witness of gene transcription, but is actively involved in multiple gene regulation processes [22], warranting further investigation. Histopathology analysis of the individual from the Portuguese family carrying this truncating mutation featured signet-ring cells as part of the tumor phenotype, as did most individuals from the Maritime sub-pedigree (except 1841, where the signet ring status was inconclusive due to lack of sufficient material).

Although *MAP3K6* mutations have not previously been identified in inherited cancer, there is a growing body of evidence that *MAP3K6* has an important role in cancer pathogenesis. In mice, where *MAP3K6* is normally expressed in gastric tissue and skin, the loss of *MAP3K6* in homozygous knockout mice was found to increase susceptibility to induced skin cancer [10]. The mice did not develop cancer spontaneously; however, chemical induction performed in the presence of an inflammatory stimulus led to a greater number of skin tumors in the *MAP3K6* deficient mice than in control animals. The number of tumors in heterozygous (*MAP3K6* ^+/−^) mice, as well as their size, was intermediate between the wild-type and knock-out mice, suggesting a role for *MAP3K6* dosage in its effects [10]. The susceptibility of these mice to gastric cancer was not assessed.

*MAP3K6* is a member of the c-Jun N-terminal Kinase (JNK) and p38 signaling cascades [14] that is most strongly expressed in the skin, gastrointestinal tract, and lungs [10]. MAP3K6 forms a heteromeric complex with its paralog MAP3K5, through their coiled-coil domains, preventing constitutive MAP3K6 degradation and promoting its autophosphorylation and activation [14]. In the presence of oxidative stress and reactive oxygen species (ROS) such as occurs following chemical (eg. DMBA) or UVA induction, active phospho-MAP3K6 activates the JNK and p38 cascades and promotes apoptosis [10], [14]. These effects have been seen in chemically induced skin-cancer models in *MAP3K6* ^−/−^ mice as well as primary keratinocyte cell lines [10].

While *MAP3K6* has a limited tissue and cell-type distribution, *MAP3K5* is more broadly expressed. The interaction of these two proteins appears to be tightly regulated, with a balance necessary between the pro-apoptotic and tumor suppressing roles of MAP3K6 and the pro-inflammatory/anti-apoptotic roles of *MAP3K5* [10], [14], [15], [23]. Expression of *MAP3K6* is variable in many human tumors, with expression most significantly reduced in gastric cancer tumors compared to healthy gastric tissue [10], whereas *MAP3K5* expression is increased [23]. Indeed *MAP3K5* ^−/−^ mice are more resistant to chemically induced gastric cancers than wild-type mice. It is clear that *MAP3K5* and *MAP3K6* are finely balanced to carry out both inflammatory and apoptotic roles, respectively in tissues where they are both expressed. Somatic mutations in *MAP3K6* have been reported in screens of a panel of 532 kinase genes in apparent non-familial gastric cancer cases (p.S291L), as well as in two gastric cancer cell lines (N87 cells: p.R375Q, and IM95 cells: p.P958T) [11]. Somatic mutations in *MAP3K6* have also been identified in other, non-gastric cancers, including ovarian (p.T968I [24]) and breast cancers (p.P869T[25], p.S648L, p.Q672* [24])). Furthermore, although expression of MAP3K6 is variable in cervical and ovarian cancer, there is a decrease in *MAP3K6* expression, compared to normal tissue, in 75% out of 106 oral, esophageal, gastric and colorectal cancer cell lines tested [10] suggesting that *MAP3K6* may have a tumor suppressive role in cancers of the gastrointestinal tract. Our results demonstrate that inherited mutations in *MAP3K6* may predispose individuals to gastric cancer; however, regulation of this pathway could have a broader role in both sporadic gastric cancers and carcinogenesis in general that warrants further investigation.

The penetrance of the disease in individuals carrying the p.P946L mutation is incomplete, with the mutation identified in 5 out of the 27 as yet unaffected individuals tested. Surprisingly, this included one individual who was homozygous for the mutation and was over the age of 80 with no reported cancer. However, there is significant variability in the age of onset (51–82 at diagnosis) in this family, unlike many other hereditary cancer syndromes, which feature an early age of onset. In particular, the average age of onset in this family is significantly higher than that typically diagnostic of HDGC. The Maritime Canadian p.P946L mutation may be hypomorphic, resulting in reduced function but not a complete loss of activity, contributing to the late age of onset and incomplete penetrance within the family, and offering a possible explanation for the individual who is homozygous for the mutation, but has not yet demonstrated signs of gastric cancer at the age of 80.

The complexity between genotype and phenotype is illustrated in Familial Adenomatous Polyposis 2 (FAP2, MUTYH-associated Polyposis), where even in recessive cancer-predisposing syndromes the penetrance and age of onset can vary significantly between pathogenic mutations within the same gene. FAP2 is a recessive disorder caused by compound heterozygous or homozygous mutations in the mismatch repair gene mutY homolog (*MUTYH*). FAP2 is characterized by an extremely elevated risk of colorectal cancer (CRC). Average age of onset of CRC in FAP2 is 48–56. Penetrance for CRC among biallelic carriers has been estimated at approximately 80% by age 80 [26] due to the presence of homozygous carriers of of the common pathogenic p.T165C (p.T179C, rs34612342) mutation in the control group of at least two case-control studies [26], [27]. This mutation is known to be pathogenic both in the homozygous state as well as a compound heterozygous mutation with other pathogenic variants. A homozygous individual for the other common FAP2 mutation, p.G396D (rs36053993), is also found in the Exome Sequencing Project (European-American) dataset. Interestingly, among CRC studies of *MUTYH* mutations while the p.T165C homozygous mutation is associated with a lower odds ratio it is generally also associated with a lower mean age of onset (48.9 versus 56.7) and more severe phenotype [28].

Incomplete penetrance of other recessive Mendelian disease is known in other cases including Leber Congenital Amaurosis [29], Schimke immuno-osseous dysplasia [30], and Bardet-Biedl syndrome [31], [32]. In those cases it is expected that the homozygous carrier of a hypomorphic allele retains more protein function than more severe combinations of compound heterozygous or the hypomorphic allele in combination with complete loss of the other allele or its expression. In addition to hypomorphism, the homozygous combination of p.P946L alleles may also be displaying over-dominance as during stress signaling *MAP3K6* molecules likely form homo-dimers as well as heteromeric complexes with *MAP3K5* [14], [33]. If the p.P946L variant impacts this self-interaction the homozygous state may confer some protection over the heterozygous state.

Alternatively, the mutation may confer risk, but as with other cancers, additional environmental and genetic factors likely play a role in the progression to gastric cancer. This is consistent with experiments in *MAP3K6* knock-out mice where cancer was only observed after the administration of both a carcinogen and an inflammatory agent, suggesting that the presence of additional stimuli are required [10]. Other variants segregating within the family may modulate both overall and individual risk of gastric cancer.

We do not know the importance of *H. pylori* in driving gastric cancer in the setting of this gene, as *H. pylori* infection was not a consistent finding among affected individuals from the Maritime Canadian family, and the rate was not different from what would be expected in the general population in this region [34], [35]. It is possible that a second, as yet unidentified genetic abnormality not detected in the exome sequencing data is also contributing to disease in that pedigree. Several regions of the genome, including in the pedigree-wide analysis, displayed significant LOD or NPL scores. The region on chromosome 1, where *MAP3K6* is located, was only identified in the reduced pedigree, where 2447 and 1845 were treated as unknown. Several regions identified pedigree-wide with high LOD scores (Table 1) are still of potential interest, and may indicate a second allele also segregating within the family contributing to the disease, with *MAP3K6* mutations modifying the phenotype in the Maritime pedigree. Regions on chromosomes 3, 7, 17, and 20 displayed consistently high LOD scores in the pedigree-wide analysis, regardless of the specified penetrance (99% or 50%) while one on chromosome 16 was identified under both models but whose LOD score was more strongly affected by the penetrance parameter.

The truncating SNV identified in the Portuguese pedigree is much more likely to result in complete loss of function of *MAP3K6*, as the frameshift and truncation occur in the region of the protein involved in activation and interaction with *MAP3K5*. While penetrance of this mutation and segregation within the pedigree is unknown, penetrance is expected to be higher than that for the p.P946L mutation. This individual was diagnosed with Hereditary Diffuse Gastric Cancer at the age of 62, intermediate between early-onset HDGC and the late-onset observed in the Maritime Canadian pedigree. The impact of the other germline variants discovered in the additional probands is not clear.

The identification of new germline mutations that appear to predispose individuals to familial gastric cancer can aid in identifying at risk individuals in affected families that are negative for *CDH1* mutations. It may also help to shed light on the underlying mechanisms leading to cancer development. Further, somatic mutations and altered expression of *MAP3K6* in sporadic cancers and gastric cancer cell lines may suggest *MAP3K6* as a potential therapeutic target for exploration. Its binding partner in the heteromeric complex, *MAP3K5*, is already being investigated for therapeutic potential [36]–[39].

The presence of *MAP3K6* mutations in the probands of six unrelated families, somatic mutations in sporadic cancers (particularly those of the gastrointestinal tract), evidence from *MAP3K6* knockout mice, second-hit mutations or hypermethylation of the wild-type allele in the tumors tested, and its molecular role in inflammation and apoptosis, all suggest that *MAP3K6* is an interesting candidate for mutations associated with Familial Gastric Cancer, warranting further study in additional cohorts of *CDH1* mutation-negative familial gastric cancer cases.

## Methods

### Ethics Statement

Approval for the research study was obtained from the IWK Health Centre research ethics board (project approval number 1005367). Informed consent was obtained from individuals or their guardians for all samples used in this study. DNA was obtained from blood, saliva or FFPE samples using standard methods

### Data Availability

Due to the small number of samples and nature of the study, genetic information can reveal potentially identifiable and unrelated health data of individuals from the family, including individuals who were not enrolled in this study. For this reason, the research ethics approval of this study and informed consent signed by participants does not allow for data to be deposited in public databases. Data used in this study are available upon request from the corresponding author pending approval from the Maritime Medical Genetics Service at the IWK Health Science Centre at: Maritime Medical Genetics Service, PO Box 9700, Halifax, Nova Scotia, Canada, B3K 6R8. Phone +1-902-470-8754.

### SNP Genotyping and Genomic Mapping

Whole genome high-density single nucleotide polymorphism (SNP) genotype scanning was performed at the McGill University and Genome Quebec Centre for Innovation, using the Illumina Human610-Quadv1_B chip (Illumina, Inc., San Diego, CA) panel with 620,901 markers and the Illumina HumanOmni 2.5M panel. DNA for SNP genotyping was isolated from either saliva or blood collected from patients. Genotype arrays were scanned using the Bead Array Reader (Illumina, Inc.), plate Crane Ex, and Illumina BeadLab software (Illumina, Inc.). Initial quality control and export of data was done using Illumina's GenomeStudio software.

Before linkage analysis was performed, the set of SNPs was pruned to obtain a subset that was appropriate for linkage analysis. Out of the 2,391,739 markers passing QC, only markers on the autosomes were retained. Markers with alleles ambiguous for strand information (A/T and G/C variants) were removed, in order to facilitate strand matching with HapMap data. Markers that were monomorphic in all genotyped samples were removed. At this point, 1,008,604 markers remained. This set of SNPs was merged with HapMap3 CEU data; only markers present in both sets of data were kept, matching on marker name. This resulted in 476,551 markers. From this set, only markers with MAF>0.4 were kept. In order to avoid inconsistencies between the observed data and the genetic map, markers with unique positions on the genetic map were selected. Arbitrarily, for a group of markers with the same genetic position, the marker with the lowest physical position was retained, and the remaining markers in the group were discarded. Finally, since linkage disequilibrium (LD) between the SNPs can arbitrarily inflate multipoint LOD scores [40], the markers were pruned to remove strong pairwise LD (r^2^<0.1 on a chromosome). This resulted in a set of 8,472 SNPs across the genome that were roughly independent, and should have high informativity for linkage analysis. For this set, the average intermarker distance was 0.57 cM, or 451 kb. Filtering was performed using a combination of PLINK v1.07 [41] and in-house scripts.

Merlin 1.1.2 [12] was used to perform multipoint non-parametric linkage on the family, using the set of 8,472 SNPs selected above. Only affected individuals will contribute to this analysis; the individuals with unknown affection status will only help infer phase. The S*_all_* statistic was used, which looks for allele sharing in all affected individuals, and tends to perform well for dominant traits [42]. The exponential model of Kong and Cox was used, which is preferred when a small number of families are analyzed [43]. CEU genotypes from HapMap3 release 28 [44] were used to estimate allele frequencies. Since it is possible that sample 2447, a distant cousin to the other affected individuals, had a slightly different phenotype, the analysis was repeated, coding this individual as having an unknown affection status, effectively removing him from the analysis. For the pedigree-wide analysis regions with an NPL score >2 were selected, with boundaries defined by markers flanking a set of SNPs with p<0.05. For the reduced pedigree with sample 2447 excluded regions with an NPL score >1 were selected.

Parametric linkage analysis was also performed, under two dominant models, one with 50% and the other with 99% penetrance. Both models used a disease allele frequency of 0.001 and 2% phenocopy rate. The analyses were performed using Merlin. Regions with LOD>1 and with SNP boundaries defined by 1 – LOD support interval were used for further analysis. Similarly to the NPL analysis above, two sets of analyses were performed with 2447 coded as either affected or unknown.

### Whole Exome Sequencing

A total of 3 µg of DNA was used for exome capture with the Agilent SureSelect All Exon 38 Mb kit. Sequencing was performed with 100-bp paired-end reads using the Illumina HiSeq 2000 at the McGill University and Genome Quebec Centre for Innovation as previously described [45]. Reads were assembled against the human genomic reference sequence (hg19) using the Burrows-Wheeler Aligner (BWA) [46]. Genomic variants were called using the Genome Analysis Toolkit (GATK) pipeline [47], and annotated with SnpEff [48] and GEMINI [49]. All variants were compared against dbSNP [50], 1000 Genomes Project [51], the Exome Sequencing Project (Exome Variant Server, NHLBI GO Exome Sequencing Project (ESP): http://evs.gs.washington.edu/EVS [Accessed October, 2013]), and a pool of exomes sequenced at the Genome Quebec Innovation Centre (1532 samples). Potentially damaging variants included non-synonymous mutations (missense and nonsense), splice-site variants, and frameshift changes due to insertions and/or deletions (indels). Exome variants were further filtered by their location in regions of the genome not excluded by linkage analysis. Variants were further selected based on their predicted impact by snpEff and GEMINI (Medium and High Impact) as well as their minor allele frequency in public databases and additional exomes sequenced at Genome Quebec (MAF< = 2%). Selected mutations in candidate genes were verified and screened in additional family members by Sanger sequencing.

### PCR and Direct Sanger Sequencing

Selected regions were amplified from genomic DNA by PCR. Amplified fragments were purified from an agarose gel, and sequenced using Sanger fluorescent sequencing and capillary electrophoresis. Sequence traces were analyzed using MutationSurveyor V.3.97 (Soft Genetics, Inc.). Because of a lack of a fresh DNA sample, initially the three children of 1884 and his spouse, 1885 were used for genotyping. The presence of the heterozygous mutation (c.[2837C>T];[ = ]) at the genomic level could be inferred for the affected individual 1884 by the absence of the mutation in his spouse, 1885, but the presence of the mutation in their child, 1903 and not in their children 1902 and 1879. The mutation was then confirmed in DNA derived from a tumorous section of an FFPE sample from this individual. An additional second-hit mutation was observed in this FFPE-derived DNA, which could be inferred to be somatic based on its absence in all three children despite both paternal copies being represented among them. In the Portuguese family, FFPE-derived DNA was extracted from the family proband's tumor (macrodissected to guarantee a minimum of 75% of tumor cells) and was used for somatic mutation analysis of the full *MAP3K6* coding sequence and intron-exon boundaries. LOH was inferred from the Sanger sequencing data, using the germline mutation site as an intragenic marker. LOH would have been considered if only the mutant allele had been found in the proband's tumor.

### Validation in an Unrelated Population

DNA previously isolated from the blood of 115 FGC patients that had been demonstrated to be negative for *CDH1* mutations were sequenced for the presence of mutations in *MAP3K6* using a multiplexed TruSeq Custom Amplicon (TSCA) sequencing assay (Illumina, San Diego). Samples were first quantified using Qubit dsDNA broad ranged assay kit (Life Technologies) and custom oligos were pooled and hybridized to individual samples. Sample indices were then added to each template library by PCR using the TSCA reagents and protocol (Illumina, San Diego). A post-PCR bead-based normalization technique was used according to the TSCA protocol to avoid the necessity of laborious and time-consuming quantification methods. Samples were then pooled prior to loading onto the MiSeq platform for simultaneous cluster generation, bi-directional sequencing and data analysis. Amplicons were designed only for coding regions (exons) of the targeted areas of interest plus up to 25 base pairs of padding around each individual amplicon. Coverage across each amplicon varied depending on location and quality of sample. In the sample where the germline *MAP3K6* frameshift variant was found, there was 80× coverage of the amplicon of interest.

### Detection of MAP3K6 Hypermethylation

We have submitted the MAP3K6 genomic sequence to a CpG island searcher (http://cpgislands.usc.edu/, [18]) and identified two CpG islands that were also annotated in ENSEMBL (release 75; www.ensembl.org). CpG island 1 was located at the promoter region and CpG island 2 encompassed exon 10 and part of the downstream intron. MAP3K6 methylation analysis was performed in the Portuguese family proband, for which FFPE tumor and peripheral blood lymphocytes (PBLs) DNA was available. Additionally, we analyzed DNA from normal stomach mucosa from controls (n = 4), and 7 gastric cancer cell lines DNA (MKN28, MKN45, NCI-N87, Kato III, AGS, GP202 and IPA220). The EpiTect Bisulfite Kit (Qiagen, Valencia, Calif) was used to treat 300 ng of DNA per sample. Unmethylated cytosines were converted to uracil, whereas methylated ones remained unmodified. A fraction of CpG sites contained within CpG islands 1 and 2 were PCR amplified using flanking primers, specifically designed for bisulfite treated DNA sequences without CpG sites, and sequenced for methylation status determination (primer sequences available upon request). Independent PCR reactions were performed at least twice for each sample.

### Predicting Pathogenicity of Detected Mutations

For all mutations detected in this work, either germline or somatic, the potential pathogenicity was predicted using a variety of published bioinformatic methods. Because individual tools are known to have differing performance profiles (both in terms of false positives and false negatives), we employed a variety of different tools that use differing algorithms for our assessment. In this work we evaluated all relevant mutations using PMut [52], PolyPhen2 [20], [53], SIFT [21], Provean [54], MutationTaster [55], EvoD [19], and FATHMM [56]. In particular FATHMM has been updated [57] with an algorithm specific to somatic mutations in cancer, which has also been used here. Unless otherwise noted default options were used with all prediction programs for their default web interface. Both PROVEAN and SIFT scores were provided through the PROVEAN interface.

## Supporting Information

Figure S1 Partial representative electropherograms from the methylation analysis of MAP3K6 CpG island 2.
(PDF)

Figure S2 Partial electropherogram showing the truncating mutation (2544delC) and the wild-type sequence at similar detection levels in the tumour from the Portuguese proband. This result allows exclusion of LOH as a second-hit, due to maintained heterozygosity at the mutation site.
(PDF)

Table S1 Summary of variants identified in the exome sequences of 1826, 1841, and 2447, located in the regions identified by parametric linkage in the entire pedigree and that met the filtering criteria described in the Methods. Variants were then sanger sequenced in other affected individuals.
(XLSX)

Table S2 Summary of variants identified in the exome sequences of 1826 and 1841 that were located in the regions identified by non-parametric linkage in the sub-pedigree represented by the proband, proband's mother, and proband's maternal aunts. Variants were filtered as described in the Methods and select variants were sanger sequenced in other affected individuals within the sub-pedigree.
(XLSX)
