# Supplementary figures and images for: Germline Mutations in MAP3K6 Are Associated with Familial Gastric Cancer
Source: PLoS Genet. 2014 Oct 23;10(10):e1004669. doi: 10.1371/journal.pgen.1004669 (PMC4207611; doi:10.1371/journal.pgen.1004669)

Supplementary figure 1.

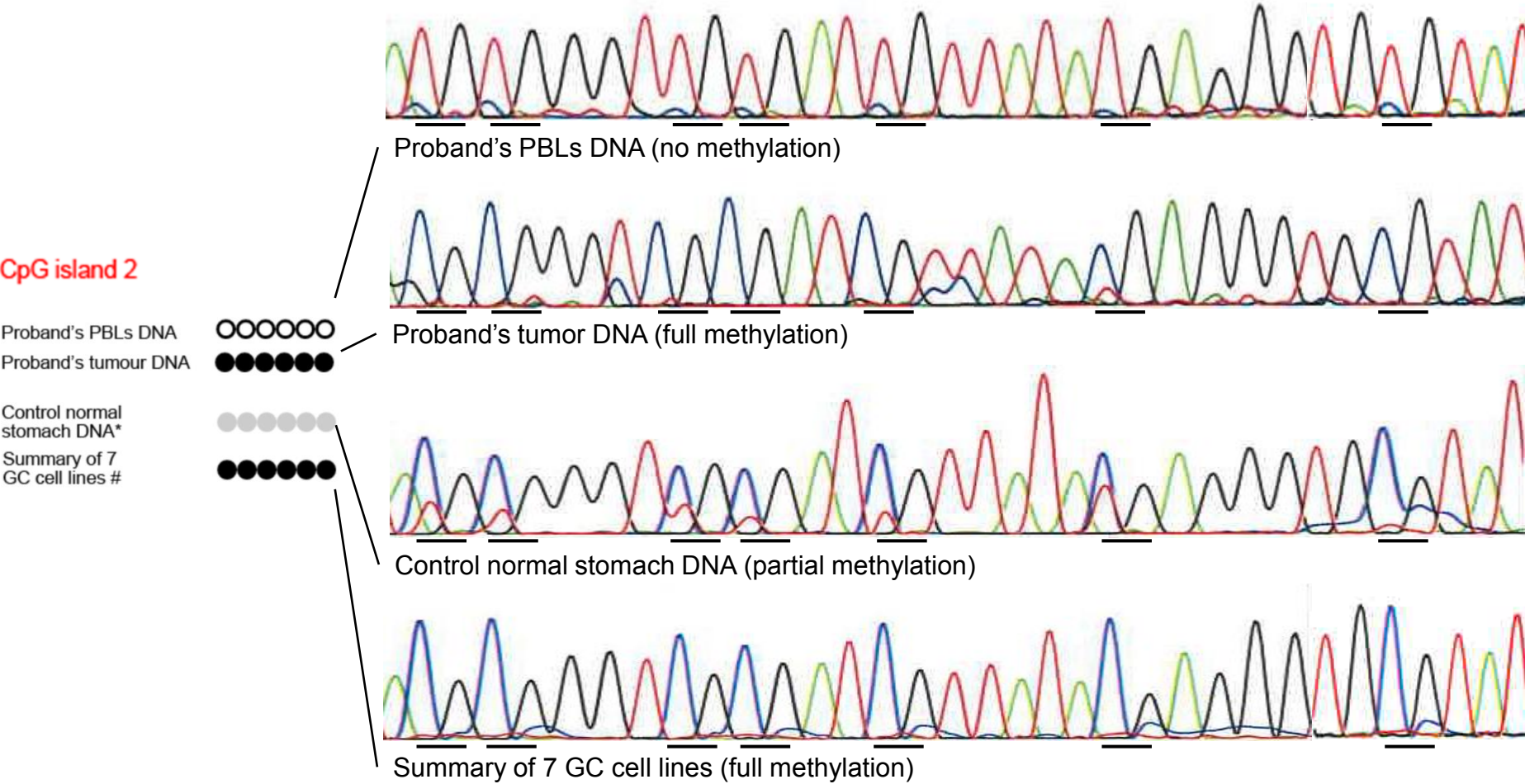

Supplement: Figure S1 — Partial representative electropherograms from the methylation analysis of MAP3K6 CpG island 2. (PDF) [file pgen.1004669.s001.pdf]
