# Supplementary material for: Germline Mutations in MAP3K6 Are Associated with Familial Gastric Cancer
Source: PLoS Genet. 2014 Oct 23;10(10):e1004669. doi: 10.1371/journal.pgen.1004669 (PMC4207611; doi:10.1371/journal.pgen.1004669)
Supplement: Figure S2 — Partial electropherogram showing the truncating mutation (2544delC) and the wild-type sequence at similar detection levels in the tumour from the Portuguese proband. This result allows exclusion of LOH as a second-hit, due to maintained heterozygosity at the mutation site. (PDF) [file pgen.1004669.s002.pdf]

Supplementary figure 2.

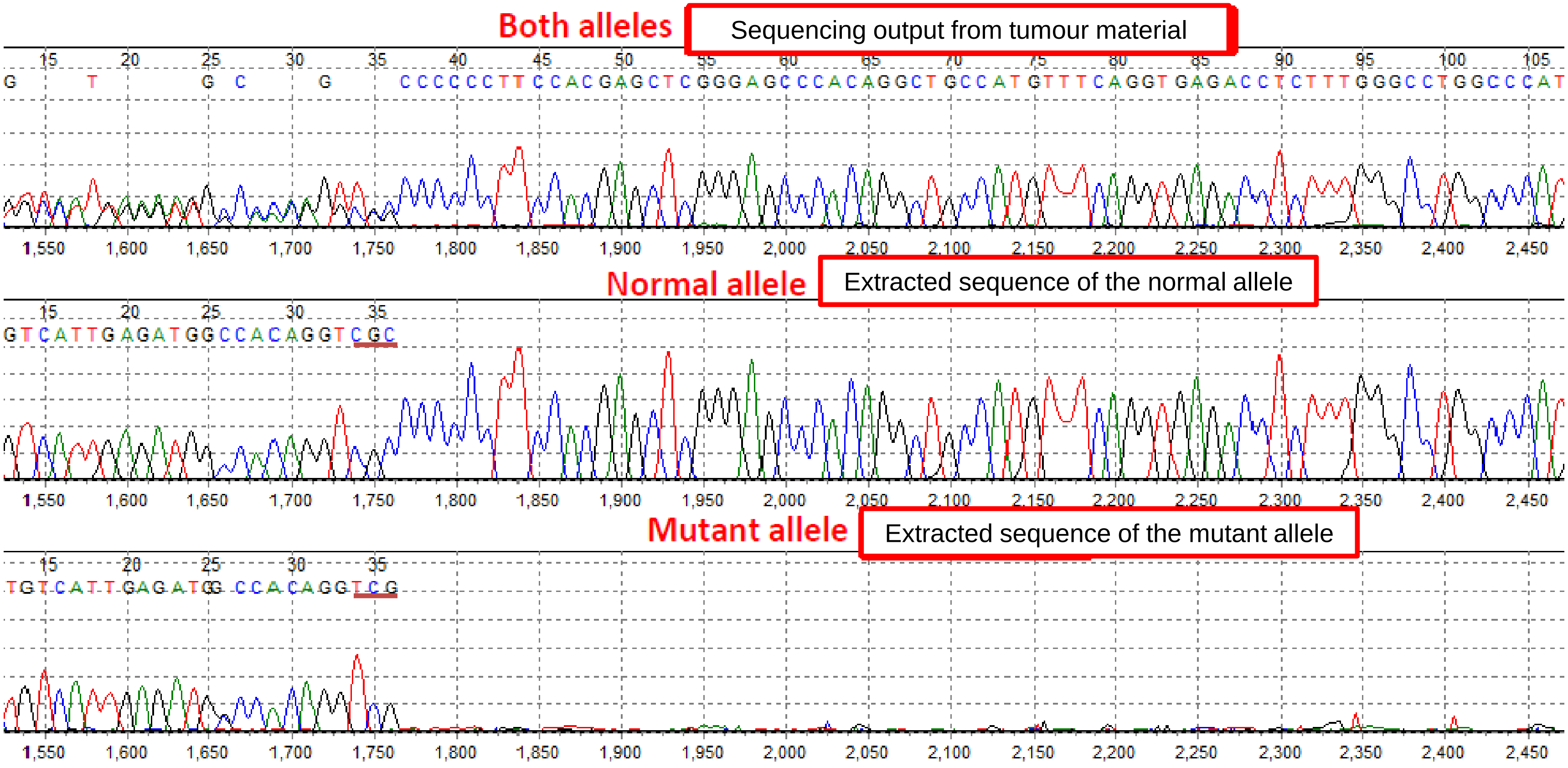

Supplementary figure 2. Partial electropherogram showing the truncating mutation (2544delC) and the wild-type sequence at similar detection levels in the tumour from the Portuguese proband. This results allows exclusion of LOH as a second-hit, due to maintained heterozygosity at the mutation site.
